# The relationship between tumour T-lymphocyte infiltration, the systemic inflammatory response and survival in patients undergoing curative resection for colorectal cancer

**DOI:** 10.1038/sj.bjc.6602419

**Published:** 2005-02-08

**Authors:** K Canna, P A McArdle, D C McMillan, A-M McNicol, G W Smith, R F McKee, C S McArdle

**Affiliations:** 1 University Department of Surgery, Royal Infirmary, Glasgow G31 2ER, UK; 2 Department of Pathology, Royal Infirmary, Glasgow G31 2ER, UK

**Keywords:** colorectal cancer, CD4+ and CD8+ T-lymphocytes, C-reactive protein, survival

## Abstract

There is increasing evidence that both local and systemic inflammatory responses play an important role in the progression of a variety of common solid tumours. The aim of the present study was to examine the relationship between tumour T-lymphocyte subset infiltration, the systemic inflammatory response and cancer-specific survival in patients with colorectal cancer. In all, 147 patients undergoing potentially curative resection for colorectal cancer were studied. Circulating concentrations of C-reactive protein were measured prior to surgery. CD4+ and CD8+ T-lymphocyte infiltration of the tumour was assessed using immunohistochemistry and a point counting technique. When patients were grouped according to the percentage tumour volume of CD4+ T-lymphocytes, there was no difference in terms of age, sex, tumour site, stage and tumour characteristics. However, there was an inverse relationship between percentage tumour CD4+ T-lymphocytes and C-reactive protein (*P*<0.01). On univariate analysis, both C-reactive protein concentrations (*P*<0.001) and percentage tumour volume of CD4+ (*P*<0.05) T-lymphocytes were associated with cancer-specific survival. The results of the present study show that low tumour CD4+ T-lymphocyte infiltration is associated with elevated C-reactive protein concentrations and both predict poor cancer-specific survival.

It has long been recognised that disease progression in cancer patients is not solely determined by the characteristics of the tumour but also by the host response. Indeed, there is increasing evidence that both local and systemic inflammatory responses play an important role in the progression of a variety of common solid tumours (O'Byrne and Dalgleish, 2001; Vakkila and Lotze, 2004).

In patients with colorectal cancer, there is good evidence that, on simple staining of tumour sections, the presence of a pronounced lymphocytic infiltration within the tumour is associated with improved survival (Jass *et al*, 1987; Ropponen *et al*, 1997; Nielsen *et al*, 1999). More recently, the ability to identify lymphocyte subsets by immunohistochemistry has led to renewed interest in the relationship between the tumour inflammatory infiltrate and outcome. Indeed, increased infiltration of the tumour by CD8+ (Naito *et al*, 1998) and CD4+ T-lymphocytes (Ali *et al*, 2004) has been shown to be associated with increased survival in patients with colorectal cancer.

There is also increasing evidence that the presence of a systemic inflammatory response, as evidenced by elevated circulating concentrations of C-reactive protein, is associated with early recurrence and poorer survival in patients undergoing potentially curative resection for colorectal cancer (McMillan *et al*, 1995, 2003; Nielsen *et al*, 2000).

To date, the inter-relationships between the local and systemic inflammatory responses and outcome does not appear to have been examined in patients with colorectal cancer. The aim of the present study was therefore to examine the relationship between tumour T-lymphocyte subset infiltration, circulating concentrations of C-reactive protein and cancer-specific survival in patients who had undergone potentially curative resection for colorectal cancer.

## PATIENTS AND METHODS

### Patients

Patients with histologically proven colorectal cancer who, on the basis of preoperative imaging and the surgeons' assessment at operation, were considered to have undergone potentially curative resection for Dukes' B and C colorectal cancer between January 1997 and August 2001 in a single surgical unit at Glasgow Royal Infirmary were included in the study. A blood sample was taken prior to surgery for the measurement of C-reactive protein. The tumours were staged using conventional Dukes' classification (Dukes and Bussey, 1958). All patients were followed up at a specialist colorectal cancer clinic.

C-reactive protein concentrations were measured by a fluorescence polarisation immunoassay and using an Abbott TDXTM analyser and Abbott reagents (Abbott Laboratories, Abbott Park, IL, USA). The limit of detection of the assay is a CRP concentration lower than 5 mg l^−1^. The coefficient of variation, over the range of measurement, was less than 5%, as established by routine quality control procedures. Based on previous work, a C-reactive protein concentration of greater than 10 mg l^−1^ was considered to indicate the presence of a systemic inflammatory response (O'Gorman *et al*, 2000).

The study was approved by the local ethics committee.

### Immunohistochemistry

Blocks from the primary tumour were fixed in 10% buffered formalin and embedded in paraffin wax. One representative block of tumour was selected for each patient. Sections (4 *μ*m) were cut and mounted on slides coated with aminopropyltriethoxysilane. Sections were then immunostained using the peroxidase-based Envision (Dako, Cambridgeshire, UK) technique as described previously (Bromwich *et al*, 2003). The primary antibody for CD4 was mouse monoclonal (Vector, Peterborough, UK) and that for CD8 was mouse monoclonal (Dako, Cambridgeshire, UK).

### Morphometry

Quantitative analysis of the lymphoid infiltrate was performed using point counting (Anderson and Dunnill, 1965) with a random sampling technique. With this method, the volume occupied by any given component (volume density) is expressed as a percentage of the total volume of the tissue. A 100-point ocular grid was used at 400 × magnification and 30 fields were counted per case for CD4+ and CD8+ immunopositive cells. Only fields within the tumour (including cancer cell nests and surrounding tissue stroma) were counted. Any normal tissue on the slide was excluded from the analysis.

This final method was designed on the basis of a pilot study, which demonstrated that the volume density of CD4+ and CD8+ of two observers reached a plateau after 25–30 fields. This pilot study also demonstrated that CD4+ and CD8+ counts were equivalent to the CD3+ counts (unpublished data). The observers (Canna and McArdle) were blinded to the clinical outcome of the patient.

### Statistics

Data are presented as median and range. Where appropriate, comparison of patient groups of patients was carried out using contingency table analysis (*χ*^2^) and the Kruskal–Wallis test for analysis of variance. For the purpose of analysis, T-lymphocyte subsets were grouped by tertiles as described previously (Nielsen *et al*, 1999). Survival analysis was performed using the Cox proportional-hazard model. Deaths up to 31st March 2004 have been included in the analysis. Analysis was performed using SPSS software (SPSS Inc., Chicago, IL, USA).

## RESULTS

The baseline clinicopathological characteristics of the patients (*n*=147) who underwent potentially curative resection for colorectal cancer are shown in Table 1. The majority were over the age of 65years and had Dukes' stage B tumours. In all, 53 (36%) patients had an elevated C-reactive protein concentration prior to surgery.

**Figure 1:**
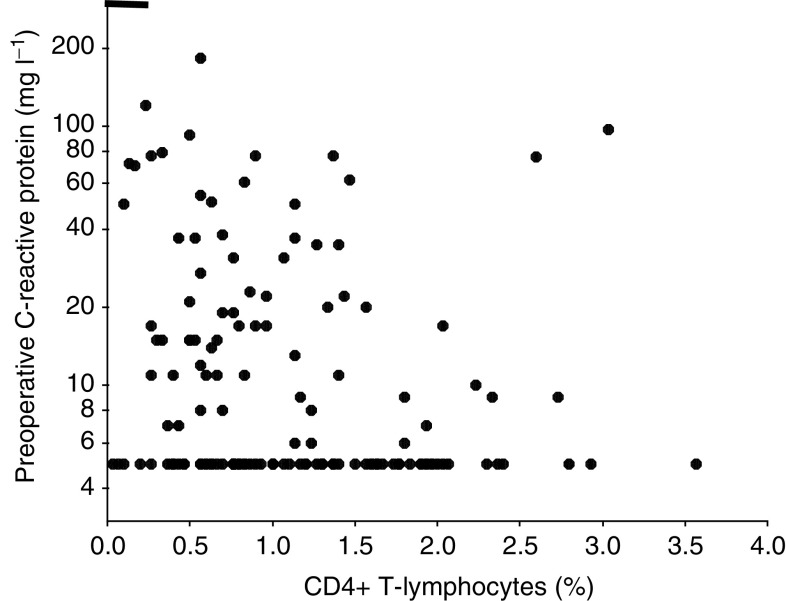
Relationship between percentage tumour CD4+ T-lymphocyte infiltration and preoperative C-reactive protein in patients undergoing potentially curative resection for colorectal cancer.

Patients grouped according to tertiles of the percentage tumour volume of CD4+ T-lymphocytes are shown in Table 2. The tertiles were similar in terms of age, sex, tumour site, stage and tumour characteristics. There was an inverse relationship between percentage tumour CD4+ T-lymphocytes and C-reactive protein (*r*_s_=−0.245, *P*=0.003, Figure 1). However, there was no relationship between percentage tumour CD8+ T-lymphocytes and C-reactive protein (*r*_s_=−0.091, *P*=0.273). There was a positive relationship between percentage tumour CD4+ and CD8+ T-lymphocytes (*r*_s_=0.440, *P*<0.001).

**Figure 2:**
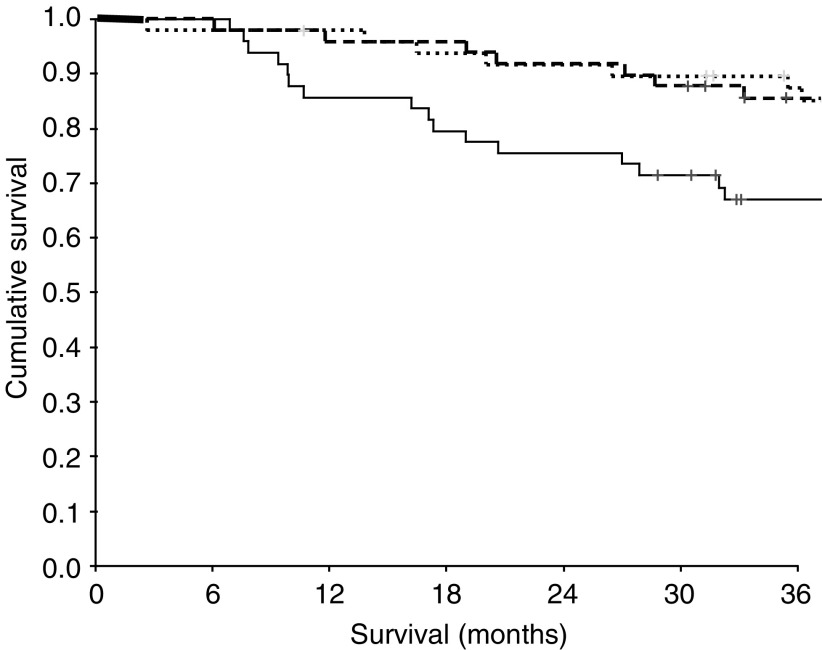
Relationship between tumour CD4+ T-lymphocyte infiltration (tertiles decreasing from top to bottom) and cancer-specific survival in patients undergoing potentially curative resection for colorectal cancer.

**Figure 3:**
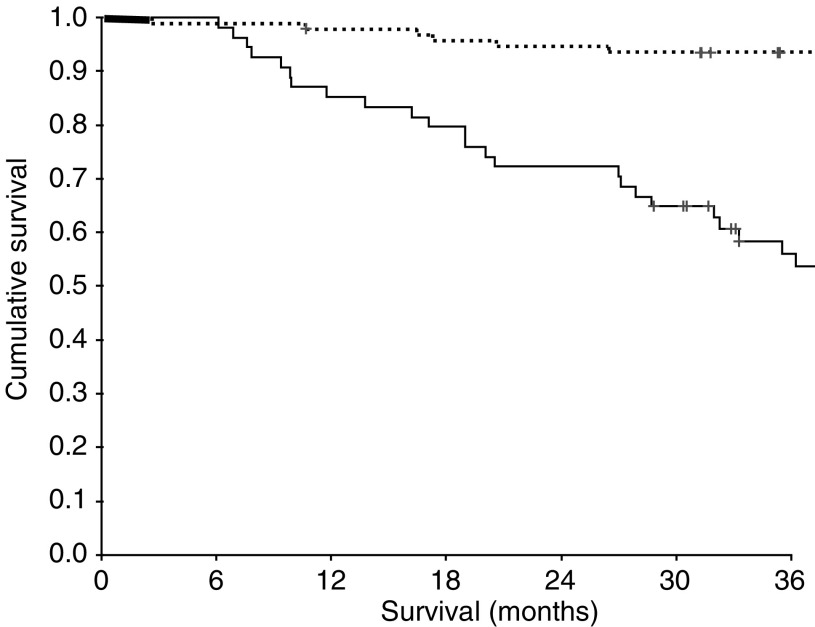
Relationship between preoperative C-reactive protein (⩽10/>10 mg l^−1^ from top to bottom) and cancer-specific survival in patients undergoing potentially curative resection for colorectal cancer.

The minimum follow-up was 30 months; the median follow-up of the survivors was 62 months. During the course of the study, 55 patients died, 39 patients of their cancer and 16 of intercurrent disease. On univariate analysis, increased age (*P*<0.001), sex (*P*=0.052), Dukes' stage (*P*<0.001) and venous invasion (*P*=0.002) were associated with poorer cancer-specific survival. A decreased percentage tumour volume of CD4+ T-lymphocytes (*P*=0.025, Figure 2) and an elevated C-reactive protein (*P*<0.001, Figure 3) were also associated with poorer cancer-specific survival. However, the relationship between percentage tumour volume of CD8+ T-lymphocytes and cancer-specific survival failed to reach statistical significance (*P*=0.074).

On multivariate analysis, only age (HR 2.05, 95% CI 1.30–3.23, *P*=0.002), stage (HR 4.39, 95% CI 2.14–9.00, *P*<0.001) and C-reactive protein (HR 4.66, 95% CI 2.20–9.89, *P*<0.001) retained independent significance.

## DISCUSSION

In the present study, a poor tumour CD4+ T-lymphocyte infiltrate was associated with an elevated circulating C-reactive protein concentration in patients undergoing potentially curative resection for colorectal cancer. Furthermore, both CD4+ T-lymphocytes and C-reactive protein were associated with a poor outcome. Therefore, the results of the present study indicate that both local and systemic inflammatory responses are linked and predict outcome independent of tumour stage.

These results appear to be consistent with previous work by Naito *et al* (1998), who showed that tumour CD8+ T-lymphocyte infiltrate had prognostic value in patients with colorectal cancer. However, they did not assess the tumour CD4+ T-lymphocyte infiltrate and used a less extensive sampling method. Furthermore, they included patients with Dukes' A tumours who were unlikely to progress and patients with Dukes' D tumours who had already progressed (Naito *et al*, 1998).

In the present study, tumour T-lymphocyte subset density was assessed using extensive sampling and a point counting technique. This approach provided an objective assessment of lymphocytic infiltration and circumvents the problem of variation in distribution of lymphocytes within an individual tumour. The present study was also confined to patients with Dukes' B and C tumours.

It was of interest that C-reactive protein (a systemic inflammatory response) was superior to tumour T-lymphocytic infiltration (a local inflammatory response) in predicting cancer specific survival. One possible explanation is that C-reactive protein can be measured with greater accuracy and precision than tumour T-lymphocytic infiltration. Alternatively, the systemic inflammatory response may be more important in determining survival in these patients.

The relationship between tumour CD4+ T-lymphocytic infiltration and cancer-specific survival is the opposite of that previously reported for both renal and prostate cancer (Bromwich *et al*, 2003; McArdle *et al*, 2004a). The reasons for this are as yet unclear. However, given that tumour lymphocytic infiltration parallels that of other inflammatory cells (Nielsen *et al*, 1999; Lin and Pollard, 2004) and that an elevated C-reactive protein is associated with poor outcome in all three tumours (Lewenhaupt *et al*, 1990; Blay *et al*, 1992; McMillan *et al*, 2003), it appears likely that the source of interleukin-6, the primary stimulus for the production of C-reactive protein, (Gabay and Kushner, 1999), differs in different tumours.

It is of interest therefore that McArdle *et al* (2004b) have recently reported that the relationship between interleukin-6 and C-reactive protein was similar in benign prostatic hyperplasia and prostate cancer, and that there was no relationship between interleukin-6 and PSA concentrations. This would suggest that, in prostate cancer at least, interleukin-6 is produced by the inflammatory cells.

In colorectal cancer, it has been reported that interleukin-6 concentrations increase with tumour stage and correlate with CEA concentrations. This might therefore suggest that interleukin-6 is produced by the tumour cells (Kinoshita *et al*, 1999; Belluco *et al*, 2000; Miki *et al*, 2004). If this were to prove to be the case, it would have important implications for the treatment of the systemic inflammatory response in patients with different tumours.

In the present study, we attempted to assess interleukin-6 within the tumour using different methods of antigen retrieval and staining and the use of negative and positive controls. However, we were unable to reliably identify regions of IL-6 expression in the colorectal tumours due to deep background staining, which precluded accurate scoring of IL-6-positive cells in the tumour tissue.

In summary, the results of the present study show that, in patients undergoing curative resection for colorectal cancer, low tumour CD4+ T-lymphocyte infiltration is associated with elevated C-reactive protein concentrations. Furthermore, both a low tumour CD4+ T-lymphocyte infiltration and an elevated C-reactive protein predict poor cancer-specific survival.

## Figures and Tables

**Table 1:** Clinicopathological characteristics in patients undergoing potentially curative resection for colorectal cancer

	**Patients (*n*=147)**
Age group (years) (<65/65–74/⩾75)	46/44/57
Sex (male/female)	78/69
Site (colon/rectum)	105/42
Dukes' stage (B/C)	91/56
C-reactive protein (⩽10/>10 mg l^−1^)	94/53

*Tumour characteristics*	
Diameter (mm)	40 (10–130)
Ulceration (no/yes)	72/75
Differentiation (well/moderate/poor)	18/116/13
Lymphatic invasion (negative/positive)	124/22
Venous invasion (negative/positive)	118/28

% *Tumour volume*	
CD4+ T-lymphocytes	0.90 (0.03–3.57)
CD8+ T-lymphocytes	1.13 (0.23–6.30)
CD4+ plus CD8+ T-lymphocytes	2.17 (0.50–8.27)

Adjuvant therapy (no/yes)	116/31
Alive/dead	92/55
Cancer-specific/intercurrent disease	39/16

**Table 2:** Relationship between increasing percentage volume of CD4+ T-lymphocytes and tumour characteristics in patients undergoing potentially curative resection for colorectal cancer

	**Percentage volume of CD4+ T-lymphocytes**			
	**Tertile (*n*=49)**	**Tertile (*n*=49)**	**Tertile (*n*=49)**	***P*-value**
Age group (years) (<65/65–74/⩾75)	12/13/24	19/11/19	15/20/14	0.122
Sex (male/female)	22/27	24/25	32/17	0.101
Site (colon/rectum)	35/14	36/13	34/15	0.905
Dukes' stage (B/C)	30/19	33/16	28/21	0.578
C-reactive protein (⩽10/>10 mg l^−1^)	22/27	32/17	39/10	0.002

*Tumour characteristics*				
Diameter (tertiles)	15/17/17	16/12/21	18/20/11	0.250
Ulceration (no/yes)	21/28	22/27	29/20	0.212
Differentiation (well/moderate/poor)	6/38/5	7/37/5	5/41/3	0.883
Lymphatic invasion (negative/positive)	40/8	43/6	41/8	0.794
Venous invasion (negative/positive)	38/10	42/7	38/11	0.554
